# The Biological Effects of 3D Resins Used in Orthodontics: A Systematic Review

**DOI:** 10.3390/bioengineering9010015

**Published:** 2022-01-03

**Authors:** Inês Francisco, Anabela Baptista Paula, Madalena Ribeiro, Filipa Marques, Raquel Travassos, Catarina Nunes, Flávia Pereira, Carlos Miguel Marto, Eunice Carrilho, Francisco Vale

**Affiliations:** 1 Faculty of Medicine, Institute of Orthodontics, University of Coimbra, 3004-531 Coimbra, Portugal; anabelabppaula@sapo.pt (A.B.P.); madalenaprata@hotmail.com (M.R.); filipa.p.s.marques@gmail.com (F.M.); raqueltravassos.91@gmail.com (R.T.); mcal9497@hotmail.com (C.N.); fppereira_@hotmail.com (F.P.); fvale@fmed.uc.pt (F.V.); 2 Faculty of Medicine, Institute of Integrated Clinical Practice, University of Coimbra, 3004-531 Coimbra, Portugal; cmiguel.marto@uc.pt (C.M.M.); eunicecarrilho@gmail.com (E.C.); 3 Faculty of Medicine, Area of Environment Genetics and Oncobiology (CIMAGO), Coimbra Institute for Clinical and Biomedical Research (iCBR), University of Coimbra, 3004-531 Coimbra, Portugal; 4 Centre for Innovative Biomedicine and Biotechnology (CIBB), University of Coimbra, 3004-531 Coimbra, Portugal; 5 Clinical Academic Center of Coimbra (CACC), 3004-531 Coimbra, Portugal; 6 Faculty of Medicine, Institute of Experimental Pathology, University of Coimbra, 3004-531 Coimbra, Portugal

**Keywords:** aligner, cytotoxicity, estrogenicity, invisalign, monomer, retainer, 3D resin

## Abstract

Three-dimensional (3D) resin medical-dental devices have been increasingly used in recent years after the emergence of digital technologies. In Orthodontics, therapies with aligners have gained popularity, mainly due to the aggressive promotion policies developed by the industry. However, their systemic effects are largely unknown, with few studies evaluating the systemic toxicity of these materials. The release of bisphenol A and other residual monomers have cytotoxic, genotoxic, and estrogenic effects. This systematic review aims to analyze the release of toxic substances from 3D resins used in Orthodontics and their toxic systemic effects systematically. The PICO question asked was, “Does the use of 3D resins in orthodontic devices induce cytotoxic effects or changes in estrogen levels?”. The search was carried out in several databases and according to PRISMA guidelines. In vitro, in vivo, and clinical studies were included. The in vitro studies’ risk of bias was assessed using the guidelines for the reporting of pre-clinical studies on dental materials by Faggion Jr. For the in vivo studies, the SYRCLE risk of bias tool was used, and for the clinical studies, the Cochrane tool. A total of 400 articles retrieved from the databases were initially scrutinized. Fourteen articles were included for qualitative analysis. The risk of bias was considered medium to high. Cytotoxic effects or estrogen levels cannot be confirmed based on the limited preliminary evidence given by in vitro studies. Evidence of the release of bisphenol A and other monomers from 3D resin devices, either in vitro or clinical studies, remains ambiguous. The few robust results in the current literature demonstrate the absolute need for further studies, especially given the possible implications for the young patient’s fertility, which constitutes one of the largest groups of patients using these orthodontic devices.

## 1. Introduction

Clear aligner systems have been around for many years in the Orthodontic practice; however, in recent years, the rapid development in this area led to the creation of large production facilities, increasing their availability to the population. With the launch of the Tooth Positioner (TP Orthodontics) in the mid-20th century, which allowed for only slight orthodontic movements, many advances were made within clear aligner systems to allow for more complex tooth movements and occlusal corrections [1,2,3]. Moreover, since Align Technology’s FDA approval in 1998, the popularity of orthodontic aligners within the general public has risen, creating a market demand and increasing the number of companies that offer these services [2,4,5,6].

These systems improved throughout the years and also retained some advantages compared to conventional orthodontic treatment. As orthodontists treat an increasingly older population, there is a rise in aesthetic concerns, favoring the use of these systems [1,2]. Another advantage is that the clear aligner systems were noted by Cardoso et al. [7] to be less painful when compared to the conventional bracket system. Reduced chair time and fewer emergencies are also listed as advantages, and treatment time seems to decrease compared to conventional systems, albeit only in mild to moderate and non-extraction cases [8,9]. Regarding oral hygiene, there is no consensual standpoint as some authors describe similar outcomes in aligner and conventional bracket patients, and others observe better hygiene and less plaque build-up in aligner patients and fewer enamel lesions as a result [3,4,5,6,8]. 

Although some of the clear aligner systems may seem to replace conventional appliances completely, these systems also present some drawbacks compared to their older counterparts [1]. Some studies indicate that clear aligner systems are less effective in controlling anterior buccolingual inclination and rotation movements in rounder teeth, and some difficulties may also be experienced to establish ideal occlusal contacts. As mentioned above, although general treatment time is decreased, in extraction cases, it is increased when compared to conventional systems [1,4,5,8,9]. As recently as 2020, concerns about the toxicity of clear aligners rose depending on their fabrication method. Studies revealed that the plastics used in clear aligner systems might have adverse effects on the activity and viability of the gingival cells and severe reproductive toxicity in an in vitro environment, highlighting potential future risks of their use in humans [10,11].

These devices are made using thermoformed, which is usually made with polyurethane with an integrated elastomer, or 3D printed plastics through different processes. Each of these manufacturing techniques presents its own set of advantages and handicaps. For example, the thermoformed method produces aligners with an irregular thickness which can create difficulties within the treatment itself. This approach is also associated with increased cytotoxicity, which is most likely due to the heating process required during the fabrication process [3,10]. As for the 3D printed plastics, these are thought to be less unsustainable and cheaper in the long run. The 3D printed plastics manufacturing method uses computer-aided design and computer-aided manufacturing technology (CAD/CAM) through additive methods (adding layers successively), subtractive methods (grinding or milling of industrially prefabricated materials), or through liquid materials (e.g., stereolithography). Although they are usually fabricated with highly cytotoxic materials such as polymethyl methacrylate (PMMA), the curing process seems to reduce this incidence but not eliminate it completely [2,3,12,13]. However, these findings are not consensual. Post processing procedures of these devices (e.g., polishing) or their sterilization (e.g., autoclaving or gamma irradiation) can also remove the uncured monomer. However, these procedures can lead to a decrease in mechanical strength. Incomplete conversion of monomers into polymers, with a marked decrease in the degree of conversion, can enhance the release of monomers, namely methyl methacrylate (MMA), triethylene glycol dimethacrylate (TEGDMA), 2-hydroxyethyl methacrylate (HEMA), and bisphenol A glycidyl methacrylate (Bis-GMA) [14,15]. These monomers can induce local negative effects such as cytotoxicity and mutagenicity, and systemic ones such as teratogenicity and estrogenicity [16,17,18]. Degradation and metabolization of these monomers can cause irreversible damage to cellular DNA. Other authors demonstrated the induction of glutathione sequestration and an increase in oxidative stress [19,20]. These phenomena can lead to changes in the cell cycle and eventually cell death by apoptosis [21,22,23]. Rogers et al. [11] reported severe reproductive toxicity after exposing murine oocytes to the materials used in clear aligner manufacturing regardless of their ISO-certification of biocompatibility or marketing ploys. On the opposite side, Eliades et al. [10] found no cytotoxicity from aligners (Invisalign) after soaking them in a saline solution for two months in a glass container set at 37 °C. These are both stand-alone research projects that have not been replicated, making it impossible for comparisons.

The purpose of this review is to assess the release of toxic substances from 3D resins used in Orthodontics and their toxic systemic effects. 

## 2. Materials and Methods

This systematic review was registered in PROSPERO with the ID 282126 number and was drawn up following the Preferring Items for Systematic and Meta-Analyses and Meta-Analyses (PRISMA) guidelines. The Population, Intervention, Comparison, and Outcome (PICO) question asked was “Does the use of 3D resins in orthodontic devices induce cytotoxic effects or changes in estrogen levels?”.

The literature search was carried out in several databases such as PubMed (www.ncbi.nlm.nih.gov/pubmed), Cochrane Library (www.cochranelibrary.com), Scopus (www.scopus.com), Web of Science Core Collection (webofknowledge.com/WOS), and EMBASE (www.embase.com). 

The last search was performed on 1 September 2021, and the language filter was applied: English, Portuguese, and Spanish. The search formula for the PubMed database was: (“3D resins” OR “3D print *” OR “invisalign” OR “Suresmile” OR “essix” OR “aligners” OR “thermoplastic aligner” OR “vacuum-formed retainer” OR “clear aligner” OR “orthodontic aligners”) AND (“bisphenol-A” OR “BPA” OR “monomer” OR “release” OR “ethoxylated bisphenol A-dimethacrylate” OR “Bis-EMA” OR “urethane dimethacrylate” OR “UDMA” OR “triethylene glycol dimethacrylate” OR “TEGDMA” OR “polyethylene glycol diacrylate” OR “PEGDA”) AND (“estrogenicity” OR estrogen OR “toxicity”[Subheading] OR toxicity[Text Word] OR “cytotoxicity”). Similar search formulas were used for the remaining databases. The references of the included studies were searched for additional relevant studies. 

Three independent reviewers scrutinized the studies by title and abstract. Potential eligible studies were selected in accordance with the defined inclusion criteria: in vitro, in vivo, ex vivo, and clinical studies; and studies evaluating the release monomers from 3D resins such as 3D printed or thermoformed orthodontic devices. Studies that only presented a chemical analysis of the aligners or considerations about the synthesis of the polymers that constitute them were excluded. Studies describing fixed orthodontic retainers with composite resins were also excluded. Two external elements were consulted in case of doubt or in the absence of consensus. 

After the eligibility process, the articles were divided into categories according to the study type: in vitro, in vivo, or clinical. For each, the following information was extracted: author and date, study design, fabrication technique, resin composition, cell line type, sample size, test group, exposure time, assay type, results, and main conclusions. In addition, for the in vivo and clinical studies, the intervention group (time of use), study measure, and the outcome data were also recorded.

The in vitro studies’ risk of bias was assessed using the guidelines for the reporting of pre-clinical studies on dental materials by Faggion Jr. [24]. For the in vivo studies, the Systematic Review Centre for Laboratory animal Experimentation (SYRCLE) risk of bias tool was used. For the clinical studies, the Cochrane tool was used.

## 3. Results

**Figure 1:**
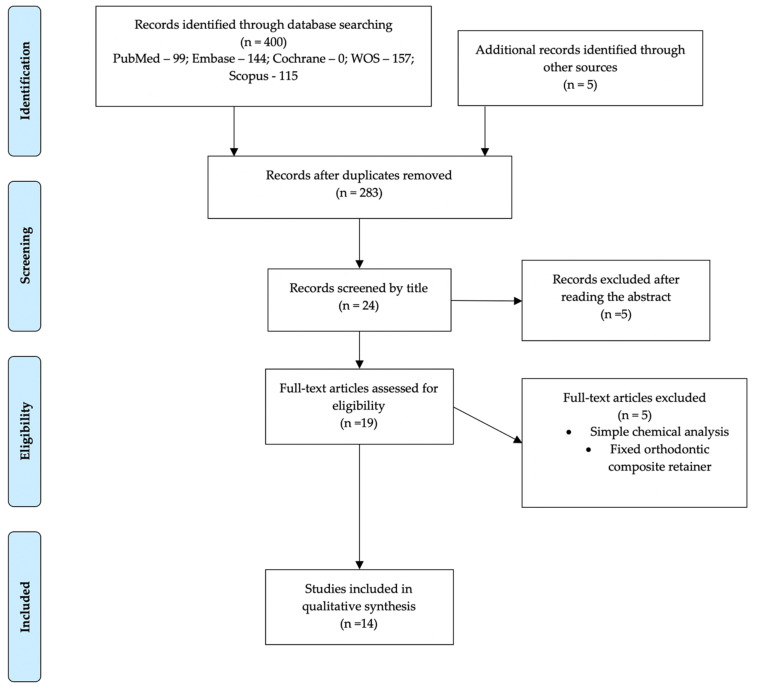
Flow Diagram of the eligibility of publication scrutiny according to PRISMA guidelines.

The search, scrutiny, and eligibility processes are described in Figure 1. The initial search resulted in 400 articles, to which five papers identified in cross-references were added. After removing the duplicates, 283 articles remained. These were scrutinized by title and abstract, resulting in 24 papers. Finally, 19 studies were read in full, and fourteen articles were included in the qualitative assessment, from which several data were analyzed, and the study of bias was carried out. The disparity of methodologies and different types of aligners/splints did not allow a quantitative analysis, so a meta-analysis was not carried out.

### 3.1. Cytotoxicity Evaluation

Of the 14 studies included in the qualitative analysis, one was a clinical study (RCT), one was an in vivo model, and the remaining 12 were in vitro studies. To assess cytotoxicity in vitro studies, those using cell lines, cell cultures, or a chemical analysis of extracts were included [10,11,25,26,27,28,29,30,31,32,33,34]. This evaluation was carried out through several assays, namely, the MTT assay, XTT assay, morphology, mass spectroscopy, gas chromatography, among others.

With the in vivo studies, the evaluation of cytotoxicity was carried out through the investigation of chemical and metallic elements in blood samples after exposure to aligners [35]. Similarly, in the included RCT, BPA levels in saliva samples from individuals exposed to aligners were assessed [36].

#### 3.1.1. In Vitro Studies

The materials used in the included studies are thermoformable resins, either printed on 3D devices or made with cold acrylics manually (Table 1). The methodology of in vitro studies is very diverse. Some studies only chemically assess the release of monomers into the medium through the extracts technique [10,25,26,28,29,30,32,33,34]. Most use these enriched media to assess their effect on cell culture through indirect contact assays. The cell lines used are mostly fibroblasts [10,27,32,33], progenitor cells such as oocytes, or estrogen sensitive cell lines [10,11,30]. Other studies also mechanically assess structural changes in aligners or retainers. Most studies report the release of monomers, especially bisphenol A (BPA), from all the devices. In some of them, these values are below the levels considered toxic. Thermoformable devices have lower monomer release values than those 3D printed, and devices made manually with heat polymerization. Another study by Alifui et al. states that only resinous materials, whether thermoformable or 3D printed, with authorization to be used for medical devices, should be recommended [34].

#### 3.1.2. In Vivo Studies

The animal study included in the systematic review evaluated some metals’ levels after using aligners or retainers in Wistar rats (Table 2). The evaluation was carried out using blood samples after several times of use. This study concluded an increase in metal levels, mainly with retainers, but they are not considered toxic. Furthermore, there is a decrease in these elements’ levels after 2 weeks. Although conducted in an animal model, this study makes an extrapolation to the levels in humans, supporting that its clinical use is safe [35].

#### 3.1.3. Clinical Studies

As mentioned above, only one clinical study evaluated the monomers’ release after retainer placement (Table 3) [36]. This analysis measured BPA levels in the patients’ saliva before placement and after 1 day, 1 week, and 1 month. All retainer types show increased levels of BPA, but only thermoformable retainers (VFRs) show statistically significant increases. Hawley retainers, either thermal or chemical polymerize, are the most recommended for clinical use.

### 3.2. Risk of Bias

**Figure 2:**
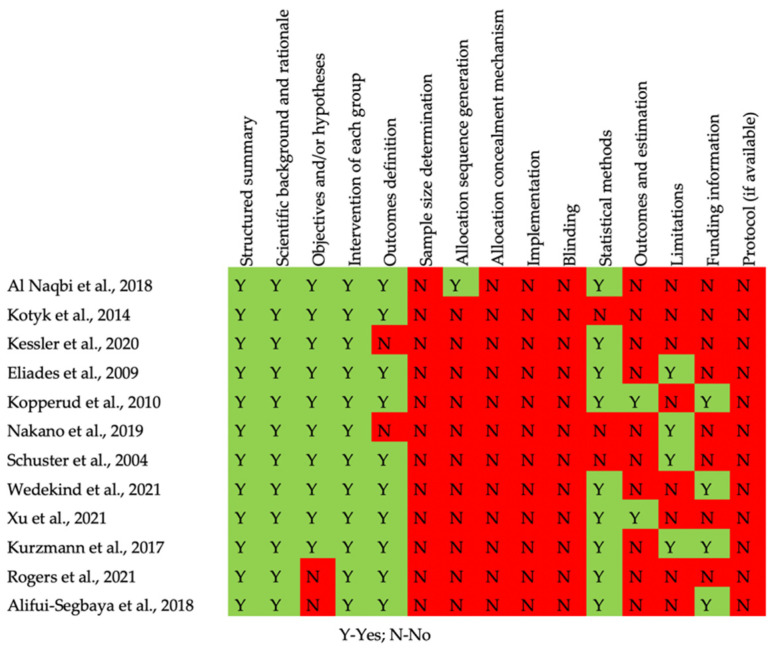
Risk of bias of the in vitro studies.

**Figure 3:**
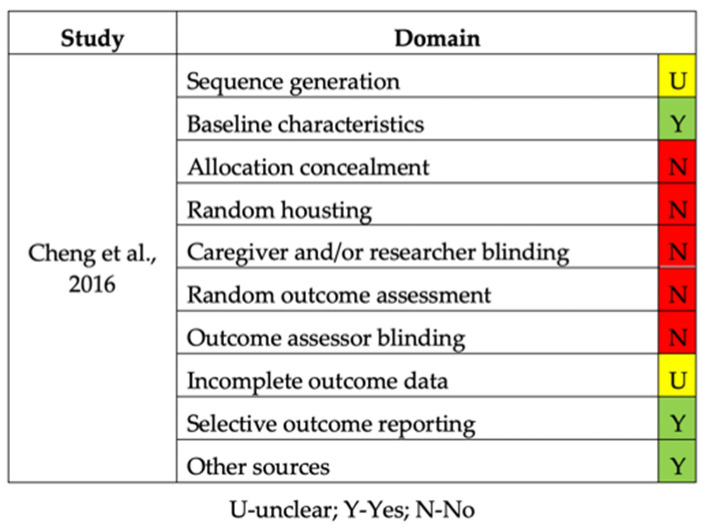
Risk of bias of the in vivo study.

The quality assessment of the in vitro and in vivo studies is summarized in Figure 2 and Figure 3, respectively. Concerning in vitro studies, only one study [30] reported the process of allocation sequence generation. All in vitro studies did not describe the mechanism used to implement the random allocation sequence, how researchers were blinded after assignment to the intervention, and where the full trial protocol can be accessed. All studies stated objectives and/or hypotheses except for two [11,34]. Regarding the in vivo study, half of the items evaluated were not presented (allocation concealment, random housing, caregiver and/or researcher blinding, random outcome assessment, and outcome assessor blinding) [35].

**Figure 4:**
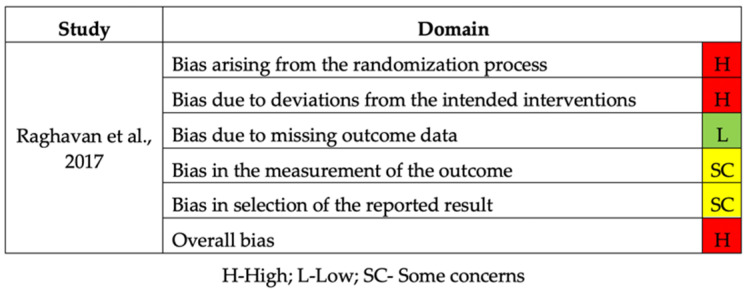
Risk of bias of the RCT study.

The included RCT was considered to have a high risk of bias due to deviations from the randomization process and intended interventions (Figure 4) [36].

## 4. Discussion

The present systematic review is set to appraise the release of toxic substances from 3D resins used in Orthodontics and its toxic systemic effects [11,13].

The development of 3D printing and advancements made in biocompatible resin materials propelled an expeditious evolution in several areas of Medicine. Nonetheless, the cytotoxic potential of 3D printed products is yet to be comprehensively researched as there is currently scarce information on this subject, and publications do not seem to reach consensual conclusions [37]. According to Eliades et al. [10], when assessing Invisalign’s cytotoxicity and estrogenicity, there were no discerning results regarding these biological effects. These results are confirmed by Iliadi et al. [37] in their systematic review of clinical and in vitro research of thermoplastic materials used in clear aligner systems, which did not report there were proven cytotoxic or estrogenic effects associated with these devices. These results agree with the findings of this systematic review. However, the qualitative report of the included studies did not allow for the establishment of a definitive consensus on the reactivity and biological properties of the clear aligners. The disparity between the results of the included studies can be explained by the methodological differences across the studies, namely the absence of sample randomization, intervention protocols, and follow-up times (varying from 24 h to 2 months). 

Kurzmann et al. [27] studied the biocompatibility and the response of the oral soft tissues to 3D printed resins. This study was set out to reveal whether 3D printed resins such as Clear Resin and Dental SG resin have an impact on human gingival fibroblasts at different processing stages. It was later concluded that the effect depends on the processing stage; in a liquid stage, the clear resin was shown to be more impactful in cell activity when compared to Dental SG resin. The in vitro study by Kopperud et al. [25] analyzed leachable monomers, additives, and degradation products from three different kinds of materials (heat-cured resin, light-cure resin, and thermoplastic) and concluded that thermoplastic materials are the least leachable out of the three.

Presently, there are concerns regarding environmental pollution promoted by the excessive use of plastics and how this affects most, if not all, ecosystems, and, in turn, how these man-made disruptions affect human health. Over time, plastic can degrade, generating micro- or nanoparticles, which leads to a plastic additives release and the absorption of environmental chemicals. This breakdown means that humans and other animals are constantly exposed to these particles through their own food or water sources, possibly causing endocrine disruption and other issues associated with plastic toxicity [38,39,40]. In addition, with the increased use of these systems and a varying number of aligners per patient, there is a rising sustainability concern, as the materials used in their fabrication are non-recyclable. Rogers et al. [11] evaluated the cytotoxic potential of 3D printed dental resins using mouse oocytes in vivo. The tested resins were Dental SG resins (DSG) and Dental LT Clear (DLT), classified as biocompatible for medical use and currently used in dental surgical guides and oral retainers. This publication concluded that although these resins are considered biocompatible, they exhibit reproductive toxicity in mouse oocytes after direct and indirect exposure. 

The articles that were included in this paper have inherent limitations. With reviews such as these, one must always account for bias risks, as different methodologies were used, assessment criteria, and accessibility to the literature. For instance, some studies compare the conventional bracket system with clear aligners with no consideration for the severity of the malocclusion nor patient cooperation, which can lead to uneven results when such studies are replicated, which is not possible many times, fueling the current replication crisis we face as investigators in a scientific field [5,9]. Furthermore, the aging of clear aligners in the oral cavity is not equal to in vitro conditions since the intra-oral environment can expose the aligners to various heat shocks, as from the ingestion of hot drinks. The oral cavity also has an alkaline environment that might promote BPA release [10]. However, identifying BPA release in clinical studies is a complex evaluation, as it requires ethical considerations.

It should also be considered that most patients using aligners are in their reproductive years. Although most of the studies report that the monomers’ release is below the toxic level, these systems require a constant change of trays, thus exposing the same individual to additive sources of BPA regularly [31].

Given the lack of literature on this subject, it is necessary to conduct further studies with similar methods focusing on the same aligner manufacturing processes and resin composition to achieve more homogeneous results, matching protocols and setting evaluation timings. Most articles published are based on in vitro studies, and despite their scientific contribution, in vivo and mainly clinical studies are required since there is increasing utilization of these systems. While further studies are not available, the orthodontists must act with caution, using the aligners for simple cases that require short treatment periods or only in non-fertile ages. In addition, orthodontists should advise patients not to ingest hot foods and instruct them on how to correctly care for the aligners to avoid the release of cytotoxic monomers.

## 5. Conclusions

Within the scope of this review, it was noted that studies evaluating the biological effects of 3D resins in orthodontics are mostly conducted in vitro. Although mixed results are described, 3D printed aligners may present higher levels of cytotoxicity and genotoxicity when compared to thermoplastic resins, particularly those that have not been subjected to a final surface treatment. As such, clinical studies analyzing saliva, blood, or even urine samples must be carried out in the future to determine the levels of monomers released in humans upon the use of these devices.

## Figures and Tables

**Table 1:** Summary of parameters and results from in vitro included studies.

Authors, Year	Study Design	Fabrication Technique	Resin Composition	Cell Line	Sample Size (n)	Test Group	Time	Assay Type	Results	Conclusions
Eliades T. et al, 2009 [10]	Extract Technique	Thermoformed	Invisalign appliances	Cytotoxicity Human gingival fibroblasts; Estrogenicity MCF-7: Estrogen-sensitivive MDA-MB-231 human breats adenocarcinoma—estrogen-insensitive.	3 sets of aligners; n = 6 (96 aligner eluents per group).	Test group: invisalign at 5%, 10%, 20%; Control group: Vehicle at 5%, 10%, 20%.	2 months	Cytotoxicity (by modification of the MTT assay); Estrogenicity (assays involved 2 cell lines: MCF-7 and MDA-MB-231).	Cytotoxicity (optical density of human gingival fibroblasts); Estrogenicity was assessed by the proliferation of MCF-7 and MDA-MB-231.	No cytotoxicity or estrogenic activity of Invisalign appliances was documented in this in vitro assay.
Kurzmann C. et al., 2017 [27]	Direct and indirect contact	Resins for Stereolithographic 3D-Printed	Clear resin (FLGPCL02), Dental SG resin (FLDGOR01)	L929 cell line, Human gingiva fibroblasts	n = 96-well culture plates	Test group: Clear (exposed to printed Clear resin) and Dental SG (exposed to Dental SG resin); Control group: W/O (untreated control).	24 h	Macroscopic and scanning electron microscopy.	When exposed to the materials, the cellular activity of L929 cells and gingival fibroblasts was observed.	The impact of Clear and Dental SG resins depends on the processing stage of the material.
Rogers H. et al., 2021 [11]	Ex vivo + in vitro (direct and indirect)	3D-printed using Form 2 SLA printers	Dental SG (DSG-FLDGOR01, Lot Nos. XN232N05, XK244N01, XK242N01, XK25N01, XH084N05) and Dental LT Clear (DLT-FLDLCL01, Lot Nos. XK484N02, XH043N02, XK29N02).	Mouse oocytes	n = 540	Test group: DSG and DLT wells; Control group: polystyrene control.	168 h	Mass spectroscopy	Exposure to DSG and DLT was proved to induce rapid mammalian oocyte degeneration in vitro.	The use of two 3DP resins revealed severe reproductive toxicity.
Kessler A. et al., 2020 [28]	Extract technique	3D-printed using Rapidshape D20 II (RS), Solflex 350 (SF), Form2 (Form).	3Delta Guide (UDMA, TMPTA, TPO); Freeprint Splint (Acrylated resin, Aliphatic urethane acrylate, TPGDA, THFMA, TPO); Fotodent Guide (BIS-EMA, Acrylresin, HEMA, HPMA, Monoester with 1,2-Propandiol, TPO); Nextdent SG (Methacrylic oligomers, Phosphine oxide); V-printed SG (BIS-EMA, UDMA, TPO).	Chemical analysis: Eluted in methanol and water for 3 days.	n = 4	Not reported	3 days	Finnigan Trace GC ultra gas chromatograph connected to a DSQ mass spectrometer.	The elution in methanol (total of twelve) and water (total of four) detected the release of substances.	The material and the printing device have a significant influence on the release of monomers from 3D-printed surgical guides.
Kotyk M. et al., 2014 [29]	Extract tecnhique	Thermoformed	Biocryl Essix (prethermoformed and thermoformed); Biocryl Retainer (prethermoformed and thermoformed); Dentsply Raintree Essix (prethermoformed), Dentsply Essix (thermoformed), Invisalign aligner (unused and used).	Chemical analysis: Eluted in artificial saliva; Bisphenol-A (BPA) leached from orthodontic materials.	n = 8 retainer materials, cut into pieces of an unspecified number.	Not reported	2 weeks	Gas chromatography/mass spectroscopy (GC-MS).	In the first 3 days of artificial saliva immersion, BPA leaching was observed.	- BPA was found to leach from thermoformed Biocryl acrylic resin retainer material; - BPA was below the reference dose for daily intake; - evidence suggests the patient BPA exposure should be minimized or even eliminated.
Naqbi A. et al., 2018 [30]	Indirect contact (extract tecnhique)	Thermoformed	Vivera retainers (from the manufacturer and after retrieved from patients).	Estrogen-sensitive MCF-7 Estrogen-insensitive MDA-MB-231.	n = 12 (6 for each of the two groups; 48 aligner eluents per group).	Test group: retainers sterilized with gamma-irradiation, retainers sterilized with autoclaving; Control group: retainers not subjected to any sterilization mode.	14 days	Cytotoxicity and Estrogenicity.	No significant proliferation of MCF-7, and MDA-MB-231 cells were induced by the three samples.	- Vivera retainers did not seem to exhibit cytotoxicity or estrogenic activity; - Vivera retainers can be used as part-time removable oral appliances following the manufacturer’s instructions.
Schuster S. et al., 2004 [31]	Mechanical test	Thermoformed	Invisalign appliances	Mechanical analysis	n = 10 samples of aligners before intraoral placement and after retrieval; n = 12 samples of same aligners after placement intraorally for 22hours for 2 weeks.	Not reported	2 weeks	Reflection microscopy, FTIR, scanning electron microscopy, Vickers hardness, Gas chromatography-mass spectroscopy (GC-MS).	Retrieved Invisalign appliance shows a morphological variation (Reflection microscopy, FTIR, scanning electron microscopy, Vickers hardness). Substance leaching (GC-MS): no residual monomers or oxidative byproducts were detected.	No definitive consensus on the reactivity and biological properties can be established.
Xu Y. et al., 2021 [32]	Mechanical test Direct contact test + extract test	Stereolithographically (SLA) printed	Dental LT Clear resin (UDMA, HEMA, EGDMA, HPA)	L929 mouse fibroblasts	n = 12	Not reported	Mechanical test—12 h Direct and indirect—12 h, 24 h, 72 h	Flexural strength test Scanning electron microscopy Metabolic activity.	No alterations were detected on the samples for less than 1 h. When post-rising prolonged to 12 h could be observed surface fissures.	The removal of cytotoxic methacrylate monomers by post rinsing could be achieved in 5 min. Further extending the post-rinsing time did not improve the cytocompatibility but rather reduced the flexural strength of the SLA-printed acrylic. - If the 3D printed material is mistakenly post-rinsed overnight(12 h), the resulting surface defects and strength reduction may not be acceptable.
Wedekind L. et al., 2021 [33]	Indirect contact	Additive manufacturing (3D-printing: SHERAprint-ortho plus); Subtractive manufacturing (SHERAeco-disc PM20); Conventional manufacturing (SHERAORTHOMER).	Polymethyl methacrylate (PMMA) (THFMA, BDDMA, TPGDA).	Human gingival fibroblasts	Not reported	Each sample eluted with water and methanol	24 h and 72 h	GC/MS analysis XTT based cell viability assay.	With the solvent methanol, the released components exceeded the cytotoxic concentrations; In water eluates, only THFMA was determined from SHERAprint-ortho plus in concentrations of non-cytotoxic levels.	With the solvent methanol, released components from the investigated splint materials exceeded cytotoxic concentrations in HGFs calculated for a worst-case scenario in splint size. In the water eluates, only the methacrylate THFMA could be determined from SHERAprint-ortho plus in concentrations below cytotoxic levels in HGFs. Therefore, in the physiological (water/saliva) situation, a health risk is of minor relevance.
Alifui-Segbaya F. et al., 2018 [34]	Indirect contact (extract tecnhique)	EnvisionTec’s digital processing (DLP) and Formlab’s reverse stereolithography (SL) systems	E-Denture (ED), E-Guard (EG), Dental SG (DSG) methacrylates.	Zebrafish embryo model	n = 10	- E-Denture (ED); - E-Guard (EG); - Dental SG (DSG) methacrylates; - control.	96 h and 120 h	FTIR spectroscopy	Biocompatibility was influenced by physicochemical characteristics of materials.	- Despite the twofold increase in DC (%) for nTx EG, it was unsafe in zebrafish bioassays; hence there is a limited correlation between conversion rate and biological performance. - The study concludes that it is preferable to use approved materials, apposite manufacturing parameters, and post-processing techniques that together ensure optimal results for medical devices.
Kopperud H. et al., 2011 [25]	Extract tecnhique	Heat-cure (Orthocryl), Light-cured (Triad VLC), Thermoplastic (Biocryl C, Essix A+, Essix Embrace) resins.	Methyl methacrylate (MMA), acetonitrile, ammonium acetate, 2,4-dinitro-phenylhydrazine (DNPH), distilled water, 2-hydroxyethyl methacrylate (2-HEMA), methanol and UDMA.	Chemical analysis: Eluted in formaldehyde	n = 5	Not reported	10 days	Gas chromatography/mass spectroscopy (GC-MS) and liquid chromatography/mass spectrometry.	Leaching methacrylate monomers from prefabricated thermoplastic plates are lower than those from powder-and-liquid-based material and from paste material.	Orthodontic prefabricated thermoplastic plates should be preferred.
Nakano H. et al., 2019 [26]	Indirect contact	3D-printed	Acrylic-epoxy hybrid light-curing resins	Not reported	n = 8	Okamoto Chemicals (3D-1M: 1); NextDent (Ortho Clear); ISO20795-2	24 h and 72 h	Cellular toxicity LDH-test Cell Viability WST1 test Mechanical experiments Stereolithography.	Have successfully developed a 3D biocompatible resin, without cellular toxicity but with not yet ideal mechanical properties.	Achieved a biocompatible 3D-printed resin that releases no toxic materials to humans or the environment.

**Table 2:** Summary of parameters and results from in vivo included studies.

Authors, Year	Study Design	Sample Size (n)	Test Groups	Fabrication Technique	Resin Composition	Outcome Time	Study Measure Outcome	Results	Conclusions
Chen S. et al., 2016 [35]	In vivo	Mini-screw implant + thermoplastic sample Wistar (n = 80).	Test group of aligner (n = 30); Test group of retainer (n = 30); Control group (n = 10); Blank group (n = 10).	Thermoformed	- Invisalign Smart Track aligners; - Erkodur retainers.	T1:28 days; T2: 56 days; T3: 112 days.	0.5 mL blood samples (rat orital vein); Inductively coupled plasma mass spectrometry (ICP-MS).	- Al, Ti, V, Cr, Fe, Co, Ni, Cu, Zn were detected in polymeric retainers; - Al, Ni, Zn, Sn were detected in polymeric aligners.	- The metal elements in polymeric materials evaluated in blood did not exceed toxic values; - The identified element levels decrease after 2 weeks.

**Table 3:** Summary of parameters and results from clinical included studies.

Authors, Year	Study Design	Sample Size (n, Sex)	Control Group	Intervention Group (Time of Use)	Fabrication Technique	Resin Composition	Outcome Time (Hours)	Study Measure Outcome	Results	Conclusions
Raghavan A. et al., 2017 [36]	RCT	n = 45: G1(n = 15); G2(n = 15); G3(n = 15. Sex: not reported	Not reported	T0: before placement; T1: 1h after placement; T2: 7 days after placement; T3: 30 days after placement.	G1: Biostar vacuum thermoforming system (VFR); G2: Hawley retainerHeat cure method; G3: Hawley retainerChemical cure method (in both arches).	G1: Essix ACE Plastic Vaccum-formed retainers (VFRs); G2: DPI Heat CureHawley retainers (compression molding technique); G3: DPI Cold Curechemical cure (“sprinkle” on technique).	180 saliva samples T0: Group 1—0.00001 ± 0.0001, Group 2—0.00006 ± 0.00004, Group 3—0.00009 ± 0.00006; T1: Group 1—1.20236 ± 0.35643, Group 2—0.00091 ± 0.00081, Group 3—0.06031 ± 0.02550; T2: Group 1—2.38420 ± 1.79714, Group 2—0.00045 ± 0.00008, Group 3—0.00363 ± 0.00050; T3: Group 1—0.020396 ± 0.08709, Group 2—0.00067 ± 0.001410, Group 3—0.00934 ± 0.00237.	BPA levels in the saliva	BPA levels: G1: T1 > T0 (+1.20 ppm); T2 > T1 (+1.18 ppm); T3 < T2 (−2.18 ppm). G2 and G3: T1 > T0; T2 < T3; T3 > T2.	- Increases BPA levels in saliva in all groups after placement of the retainers; - BPA levels were found to be larger in VFRs, followed by Hawley retainers by chemical cure, and finally Hawley retainers by heat cure; - VFRs increase BPA levels after placement to 1 week but decrease after 1 month; - Hawleys retainers (heat and chemical) decrease after placement to 1 week but increase after 1 month.

## Data Availability

The data presented in this study are available on request from the corresponding author.
